# Effects of Short Video App Guided Mindfulness Meditation on Policemen’s Communication Anxiety, PTSD, Anger Management, and Mood Disorders

**DOI:** 10.3390/healthcare13101213

**Published:** 2025-05-21

**Authors:** Chao Liu, Li-Jen Lin, Kang-Jie Zhang, Thu-Hua Liu, Wen-Ko Chiou

**Affiliations:** 1 School of Journalism and Communication, Hua Qiao University, Xiamen 361021, China; victory666666@126.com; 2 General Education Center, Mindfulness Meditation Center, Ming Chi University of Technology, New Taipei 243303, Taiwan; llien@mail.mcut.edu.tw; 3 School of Education, City University of Macau, Macau, China; m24091100642@cityu.edu.mo; 4 Department of Industrial Design, Ming Chi University of Technology, New Taipei 243303, Taiwan; thliu@mail.mcut.edu.tw; 5 Department of Industrial Design, Chang Gung University, Taoyuan 33302, Taiwan

**Keywords:** mindfulness meditation, police officers, PTSD, communication anxiety, anger

## Abstract

Background: Law enforcement is a high-stress profession, with officers frequently exposed to traumatic events, leading to mental health challenges such as communication anxiety, post-traumatic stress disorder (PTSD), anger management difficulties, and mood disorders. Mindfulness meditation (MM), particularly when guided through short video applications, has shown promise in addressing these issues by enhancing emotional regulation and resilience. Objective: This study explores the effects of an 8-week MM intervention, delivered via short video apps, on communication anxiety, PTSD, anger management, and mood disorders in police officers. Methods: A randomized controlled trial (RCT) was conducted with 110 full-time police officers aged 25–55 in China. The final 92 eligible participants were divided into two groups: the MM group (*n* = 46) and the control group (*n* = 46). The intervention consisted of daily 10–15 min video-guided MM sessions. Pre- and post-intervention measures included validated questionnaires assessing communication anxiety (PRCA-24), PTSD (PCL-5), anger management (STAXI-2), and mood disorders (DASS-21). Data analysis was performed using MANOVA. Results: The intervention group showed significant improvements in communication anxiety (F = 8.505, *p* = 0.004), PTSD (F = 25.831, *p* < 0.001), anger management (F = 4.968, *p* = 0.027), and mood disorders (F = 13.058, *p* < 0.001) compared to the control group. These improvements were supported by significant interaction effects between group and time, indicating that the MM intervention had a positive impact on these mental health variables. Conclusions: Video-guided MM delivered via short video apps significantly reduced communication anxiety, PTSD symptoms, and mood disorders, and improved anger management among police officers. These findings highlight the potential of digital MM interventions as a scalable and accessible tool for enhancing mental well-being and resilience in law enforcement personnel.

## 1. Introduction

Police officers operate in a high-stress, high-risk work environment that exposes them to numerous psychological challenges, including communication anxiety, post-traumatic stress disorder (PTSD), anger management difficulties, and mood disorders [1]. The nature of police work involves frequent exposure to violent incidents, high-pressure decision-making, and emotionally charged interactions, all of which contribute to long-term mental health concerns [2].

Extensive research has documented the prevalence of mental health issues among law enforcement personnel. For example, studies estimate that 15% to 35% of police officers globally exhibit PTSD symptoms [3]. Additionally, rates of anxiety and depression among police officers are significantly higher than those observed in the general population [4]. However, beyond PTSD, anxiety, and mood disorders, police officers also experience high rates of substance misuse and elevated suicide risk, both of which warrant serious attention. Research indicates that police officers are significantly more likely to engage in alcohol misuse compared to civilians, with alcohol abuse rates estimated at 25–30% [5]. Furthermore, police officers have a higher suicide rate than many other occupational groups, particularly due to PTSD, chronic occupational stress, and limited access to mental health resources [6].

Despite the widespread prevalence of these issues, police officers often face significant barriers to seeking psychological support. Traditional mental health interventions, such as face-to-face counseling or pharmacological treatments, may not be feasible due to time constraints, confidentiality concerns, and stigma associated with seeking psychological help in law enforcement cultures [7]. Consequently, alternative non-pharmacological, accessible, and stigma-free interventions are needed to address these challenges effectively.

One promising intervention is mindfulness meditation (MM), which has been increasingly recognized for its ability to reduce stress, improve emotional regulation, and enhance resilience in high-risk professions [8]. Studies suggest that mindfulness-based interventions can alleviate symptoms of PTSD, anxiety, and depression while also fostering greater self-awareness and emotional balance [9]. Given the potential benefits of mindfulness, this study examines the effectiveness of short video app-guided mindfulness meditation (SVA-MM) as an intervention to improve psychological well-being and interpersonal functioning among police officers. Specifically, this study focuses on how SVA-MM influences communication anxiety, PTSD symptoms, anger regulation, and mood disorders, while acknowledging the need for future research to explore its impact on substance misuse and suicide risk within law enforcement settings.

Recent systematic reviews and meta-analyses have demonstrated that digital delivery of MM programs via mobile applications, video platforms, and online modules is effective in reducing symptoms of anxiety, depression, and stress across diverse populations) [10,11]. Compared to in-person delivery, app-based MM offers greater accessibility, user autonomy, and anonymity, which are particularly advantageous in high-stigma environments such as law enforcement. One meta-analysis found that self-guided digital mindfulness interventions produced small to moderate effects on psychological outcomes, with enhanced adherence when programs included short video content and guided instructions [12]. This growing body of evidence supports the viability of short-video app-guided mindfulness interventions as both effective and scalable solutions for occupational mental health support. In light of these findings, we adopted a short video app as our delivery modality to ensure high accessibility, consistent engagement, and cultural acceptability for our target population of Chinese police officers.

### 1.1. The Growing Relevance of Mindfulness Meditation in Policing

Mindfulness meditation (MM), a form of mental training focused on cultivating present-moment awareness and emotional regulation, has shown promise as an effective tool for managing stress and improving mental health outcomes across diverse populations [13]. Notably, MM may also reduce cognitive biases toward negative information, a critical adaptation for law enforcement professionals who routinely encounter high-threat environments. For instance, long-term meditators (e.g., Shaolin monks) exhibited reduced attentional bias to anger stimuli compared to non-meditators, suggesting that mindfulness fosters impartial processing of emotionally charged stimuli [14]. Similarly, mindfulness interventions for chronic pain patients decreased pain-related attentional bias, which was linked to improved emotional regulation and perceived control over distress [15]. MM encourages individuals to focus on their current experiences, thoughts, and feelings non-judgmentally, fostering greater emotional awareness and the capacity to regulate reactions to stressors. For police officers, who regularly navigate stressful environments, MM has the potential to enhance their emotional resilience, which could mitigate the impact of work-related stressors on mental health [16]. Law enforcement personnel are at heightened risk for a variety of mental health disorders due to occupational stress, exposure to trauma, and shift work. Studies indicate that 20–30% of police officers experience PTSD symptoms, while substance misuse rates among officers are higher than in the general population, often as a maladaptive coping mechanism for stress [17]. Additionally, suicide rates among police officers are disproportionately high, with research suggesting that law enforcement personnel may be 54% more likely to die by suicide compared to the general workforce [18]. Despite these risks, many officers do not seek mental health services due to stigma, fear of professional repercussions, and lack of accessible care [19]. Given these barriers, scalable, stigma-free interventions such as mindfulness meditation delivered via digital platforms may provide an accessible means for officers to manage mental health challenges proactively.

The accessibility of MM has been further facilitated by the growth of digital technology, particularly short video applications. Short video apps enable easy access to mindfulness sessions, allowing users to engage with MM practices at convenient times and locations, thus overcoming some logistical barriers traditionally associated with in-person interventions [8]. Given the adaptability of short video-based MM interventions, police officers can integrate mindfulness practices into their daily routines more seamlessly. However, in addition to logistical constraints, police officers face substantial barriers in seeking mental health services due to cultural and professional factors. Policing culture often values toughness and emotional control, which discourages officers from acknowledging mental health concerns or seeking professional help [20]. Many officers worry about the potential negative impact on their careers, as seeking psychological support might be perceived as a sign of weakness or professional incompetence [21]. Moreover, police officers may harbor distrust toward service providers who lack experience in law enforcement-related trauma [22]. These challenges create a significant gap between mental health service availability and actual utilization.

In recent years, digital mental health interventions have been increasingly applied to high-stress occupations. For example, Flett et al. (2019) [23] demonstrated that a smartphone-based mindfulness app reduced burnout and emotional exhaustion among frontline healthcare workers. Similarly, Wielgosz et al. (2024) [24] reported significant improvements in PTSD and depression symptoms among veterans using mobile-delivered cognitive-behavioral interventions. Cameron et al. (2025) [25] conducted a systematic review and found strong preliminary support for the use of digital interventions—including mindfulness, telehealth therapy, and mobile support apps—for emergency responders facing chronic stress and trauma exposure.

These studies highlight the growing effectiveness and acceptance of digital mental health solutions in high-pressure professions. However, evidence from non-Western contexts and from law enforcement personnel remains limited. Our study addresses this gap by testing a culturally adapted, video-guided mindfulness meditation program in a sample of Chinese police officers—a group with unique occupational stressors and barriers to psychological support. By situating our work within this expanding field of digital intervention research, we underscore the broader applicability and value of short-video-based mindfulness tools for first responders.

### 1.2. Barriers to Mental Health Service Utilization Among Police Officers

Despite the recognized need for mental health interventions among police officers, multiple barriers prevent them from seeking professional support. Beyond logistical constraints such as scheduling conflicts and limited availability of in-person services, psychological and cultural barriers significantly contribute to low utilization rates. The law enforcement profession often fosters a culture of self-reliance and emotional suppression, discouraging officers from openly discussing mental health issues. Officers may fear being stigmatized by peers or supervisors, which could impact their career advancement or lead to perceptions of incompetence [22]. Additionally, concerns about confidentiality and mistrust toward mental health professionals further discourage help-seeking behaviors [21].

Digital mental health interventions, such as mindfulness-based programs delivered through smartphone applications, offer a promising alternative for overcoming these barriers. Mobile-based interventions allow police officers to engage in mental health practices privately and discreetly, reducing stigma-related concerns. These interventions also provide flexibility in scheduling, enabling officers to integrate mindfulness training into their daily lives without disrupting their professional responsibilities [20]. By leveraging a short-video app format, this study explores how mindfulness-based interventions can serve as a stigma-free and accessible tool for addressing key mental health concerns among police officers.

### 1.3. Theoretical Underpinnings of Mindfulness Meditation Effects on Mental Health

Several psychological theories explain the mechanisms through which MM can positively impact mental health outcomes. Self-Determination Theory (SDT) suggests that MM enhances intrinsic motivation and supports autonomy, fostering greater emotional resilience and self-regulation [26]. By cultivating non-reactivity and self-awareness, MM enables individuals to manage their emotional responses more effectively. Social Learning Theory (SLT), which emphasizes the role of observation and imitation in learning, further supports the potential of video-guided MM to promote mental health benefits. Through observation of MM practices, police officers can learn and model effective emotional regulation strategies, which may help in managing anxiety, PTSD symptoms, and other mood disturbances [27].

### 1.4. Mindfulness Meditation (MM) and Communication Anxiety

Communication anxiety, a common challenge in high-stress occupations such as policing, refers to the fear or apprehension associated with actual or anticipated communication with others [28]. This type of anxiety can impact officers’ abilities to communicate effectively, leading to strained interpersonal relationships and potentially hindering their performance on the job. For police officers, effective communication is essential not only for interacting with the public but also for maintaining positive relationships with colleagues and handling complex situations with clarity. Mindfulness meditation (MM), which emphasizes present-moment awareness and non-judgmental observation of thoughts and emotions, has been shown to help manage communication anxiety by promoting emotional regulation and reducing automatic stress responses [29].

MM aids in reducing communication anxiety by fostering a detachment from distressing thoughts and physiological responses. Research indicates that mindfulness practices encourage individuals to observe their anxious thoughts without engaging or reacting impulsively. For instance, a study by Goldin and Gross [29] found that participants who practiced MM reported lower levels of social anxiety and exhibited less avoidance in communicative contexts. By training individuals to observe their thoughts and emotions as temporary, MM allows individuals to manage communication scenarios without becoming overwhelmed by fear or stress [30].

In high-stress environments like policing, where communication often occurs under pressure, MM can serve as a practical tool for emotional regulation. Techniques such as focused breathing and awareness exercises enable police officers to calm their physical reactions, such as elevated heart rate and sweating, which are common responses to anxiety [31]. Through consistent practice, officers can gain a sense of control over their physiological responses, allowing them to communicate with greater confidence and effectiveness. Additionally, MM fosters self-compassion and self-acceptance, which are associated with reduced self-criticism—a factor that often exacerbates communication anxiety [32].

The digital delivery of MM through short video applications adds accessibility and convenience, making it easier for officers to integrate mindfulness practices into their busy schedules. Research on digital mindfulness interventions has shown positive effects in reducing anxiety across various settings. For example, Trombka et al. [33] found that short video-based mindfulness practices improved self-reported communication skills and decreased anxiety, providing a viable model for law enforcement professionals. The combination of emotional regulation and ease of access positions MM as a valuable resource for reducing communication anxiety in policing, with the potential to enhance both personal well-being and professional efficacy.

### 1.5. Mindfulness Meditation and Post-Traumatic Stress Disorder (PTSD)

Post-traumatic stress disorder (PTSD) is a prevalent and serious mental health issue among law enforcement officers, who often encounter traumatic events as part of their work. PTSD symptoms, such as intrusive thoughts, hypervigilance, avoidance behaviors, and emotional numbing, can interfere with daily functioning and the ability to perform job duties effectively [34]. These symptoms are particularly detrimental in high-stress professions like policing, where emotional resilience and mental clarity are essential. Mindfulness meditation (MM) has gained recognition as a promising intervention for reducing PTSD symptoms through mechanisms that include emotional regulation, present-moment focus, and cognitive reframing [35].

One of the primary ways MM helps alleviate PTSD symptoms is by enhancing emotional regulation. Mindfulness encourages practitioners to focus on their present experiences and observe their thoughts and emotions without reacting impulsively. Through regular MM practice, individuals develop a non-reactive awareness, which can help them better manage distressing memories and emotional triggers associated with trauma [36]. This aspect of MM is particularly beneficial for police officers who are at risk of re-experiencing traumatic memories in high-stress situations. By learning to accept and observe these memories non-judgmentally, MM can reduce the likelihood of retraumatization and help officers maintain composure in challenging scenarios [37].

Additionally, MM has been shown to reduce PTSD symptoms by decreasing rumination and hyperarousal. Rumination, a common characteristic of PTSD, involves repeatedly dwelling on negative thoughts and past events, which can worsen symptoms. Through MM practices, individuals cultivate an awareness that allows them to recognize and disrupt these repetitive, distressing thought patterns. In a study by Davis et al., participants with PTSD who engaged in mindfulness meditation reported reduced rumination, as well as decreased hyperarousal and avoidance behaviors [38]. These findings suggest that MM can improve cognitive processing of traumatic memories, allowing individuals to approach them with a balanced and detached perspective.

Moreover, MM provides a structured approach to safely process traumatic memories. By creating a mental space where individuals can confront painful memories without becoming overwhelmed, MM promotes a sense of psychological safety and resilience. For example, Ledoux et al. [39] found that MM significantly reduced PTSD symptoms in military personnel by providing a mental framework to process trauma non-judgmentally, leading to better emotional regulation. This is crucial for police officers, who may not have the time or resources to attend lengthy therapy sessions. Video-based MM interventions, which can be accessed as needed, offer an accessible alternative that fits the demanding schedules of law enforcement professionals.

The growing evidence supporting MM’s benefits in treating PTSD has also highlighted its adaptability through digital platforms. Short video app-guided MM interventions allow officers to engage in mindfulness practices at their convenience, increasing accessibility and consistency of use. This flexibility is especially valuable in law enforcement settings, where scheduling constraints can limit participation in traditional, in-person interventions. By leveraging digital technology, MM can provide police officers with an on-demand tool for managing PTSD symptoms, fostering resilience, and enhancing overall mental health in the face of ongoing stress and trauma [8].

### 1.6. Mindfulness Meditation and Anger Management

Anger management is essential for police officers, who often encounter high-stress, potentially confrontational situations that demand emotional control and measured responses. Uncontrolled anger can lead to impulsive decisions and reactions, which may not only jeopardize officers’ safety but also strain community relations [40]. Mindfulness meditation (MM) has gained recognition for its ability to help individuals manage anger by fostering self-awareness, patience, and emotional regulation. By training practitioners to observe and detach from intense emotions, MM creates a mental buffer that enables more constructive responses to anger-provoking situations [41].

MM techniques focus on enhancing present-moment awareness, allowing practitioners to recognize early signs of anger without immediately reacting. When police officers practice MM, they develop an ability to notice physiological and emotional indicators of anger, such as increased heart rate or irritability, and use breathing or awareness exercises to de-escalate these reactions. This proactive awareness is crucial for preventing anger from escalating into impulsive actions. Research supports the efficacy of MM in anger management, with findings indicating that regular mindfulness practice helps individuals detach from automatic reactions and promotes emotional resilience. For example, Wright et al. [42] found that MM reduced the frequency and intensity of anger episodes by fostering an attitude of non-reactivity and promoting emotional stability.

The benefits of MM in anger management extend to various high-stress professions. A study by Bailey [31] observed that participants in a high-stress occupational group who underwent MM training exhibited significant reductions in both the intensity and frequency of anger episodes. This decrease in reactive anger is attributed to MM’s role in training individuals to observe their thoughts and feelings with acceptance, thus reducing the likelihood of impulsive or aggressive behavior. By promoting patience and empathy, MM can empower police officers to handle volatile situations calmly and effectively [29].

In addition to emotional regulation, MM encourages a compassionate perspective that can further support anger management. MM fosters a mindset that values empathy and understanding, both of which contribute to non-violent conflict resolution. For police officers, this aspect of MM practice can be instrumental in transforming their approach to anger-provoking scenarios, allowing them to view interactions with empathy rather than react with frustration or aggression. Studies indicate that MM’s emphasis on kindness and compassion may enhance officers’ ability to manage anger by helping them reframe challenging situations more constructively [43].

Furthermore, digital delivery of MM through short video applications offers a convenient and accessible means for officers to incorporate mindfulness into their daily routines, which can be challenging in their demanding work environments. Video-guided MM sessions allow for consistent practice, enabling officers to develop skills for noticing and managing anger in real time. By integrating MM into policing through digital platforms, law enforcement agencies may effectively equip officers with tools to regulate their emotions and approach potentially volatile encounters with composure, ultimately contributing to safer and more positive interactions with the public [44].

### 1.7. Mindfulness Meditation and Mood Disorders

Mood disorders, including depression and generalized anxiety, are prevalent among law enforcement officers who experience frequent exposure to high-stress situations and traumatic events. These disorders can lead to significant emotional distress, impaired functioning, and decreased job satisfaction [45]. Mindfulness meditation (MM) has been studied extensively as a therapeutic intervention for mood disorders, with research indicating its effectiveness in enhancing emotional regulation, reducing stress, and promoting overall psychological well-being. By fostering present-moment awareness and minimizing rumination, MM can alleviate symptoms associated with mood disorders and provide law enforcement officers with a sustainable tool for managing their mental health [46].

MM addresses mood disorders by enabling individuals to observe their thoughts and emotions without judgment. This non-judgmental awareness helps individuals disengage from negative cognitive patterns, which are often characteristic of depression and anxiety. For instance, Lu et al. [47] found that MM significantly reduced depressive symptoms in participants by fostering a more balanced and accepting approach to negative thoughts and feelings. Rather than becoming entangled in these thoughts, practitioners learn to view them as transient experiences, reducing the impact of depressive rumination. For police officers, who are prone to the cumulative stress of their work, this shift in perspective can contribute to improved emotional resilience and reduced susceptibility to mood disorders [48].

Physiologically, MM has been shown to lower stress markers, such as cortisol, which is frequently elevated in individuals with mood disorders due to chronic stress exposure. By decreasing cortisol levels and enhancing parasympathetic nervous system activity, MM promotes relaxation and mood stability [8]. This physiological response not only alleviates symptoms of anxiety and depression but also reinforces a sense of calm and emotional stability, both essential for effective functioning in high-stress environments like law enforcement. Regular MM practice supports the development of a relaxed yet alert state, allowing police officers to engage with their work without the excessive emotional burden often linked to mood disorders [49].

The accessibility of MM through short video applications further enhances its potential as a practical intervention for law enforcement personnel. Digital platforms make MM exercises available at any time, accommodating the demanding schedules typical in policing. Moreover, the ability to access MM training anonymously through a mobile app may encourage greater engagement among officers who might otherwise hesitate to seek mental health support due to concerns about stigma or career repercussions. By offering a private, self-guided approach to emotional regulation, video-based MM interventions align with the unique cultural and professional constraints faced by law enforcement personnel [50]. Flett et al. [23] found that mobile app-based MM practices not only improved accessibility but also encouraged more consistent practice, thereby amplifying the intervention’s benefits. With the ability to incorporate MM into daily routines, officers have access to an effective, time-efficient tool for managing mood disorders. By promoting emotional balance and stress reduction, app-based MM interventions provide a valuable resource for enhancing officers’ mental health and well-being [51].

The evidence supporting MM for managing mood disorders highlights its potential to improve the mental health outcomes of police officers, leading to better job satisfaction and performance. By reducing depressive symptoms, stabilizing mood, and fostering a resilient mindset, MM enables officers to meet the emotional demands of their role more effectively. In professions where exposure to stress and trauma is inevitable, MM provides a proactive approach to mental healthcare, ensuring officers are better equipped to cope with the challenges of their work [52].

This study explores the effectiveness of mindfulness meditation guided by short video apps in addressing key mental health challenges faced by police officers. By investigating the impact of MM on communication anxiety, PTSD, anger management, and mood disorders, this research aims to provide valuable insights into how digital mindfulness interventions can support mental well-being and resilience among law enforcement personnel. The study hypotheses propose that MM interventions will reduce communication anxiety, alleviate PTSD symptoms, improve anger management, and decrease mood disorder symptoms in participants. These findings could have meaningful implications for developing accessible mental health support tailored to the unique demands of policing.

**Hypothesis** **1** **(H1).** *MM intervention will significantly reduce communication apprehension, as measured by the Personal Report of Communication Apprehension (PRCA-24).*

**Hypothesis** **2** **(H2).** *MM intervention will significantly reduce PTSD symptom severity, including intrusive thoughts, hypervigilance, and avoidance behaviors, as measured by the PTSD Checklist for DSM-5 (PCL-5).*

**Hypothesis** **3** **(H3).** *MM intervention will significantly reduce anger expression and increase anger control, as measured by the Short Form of the State-Trait Anger Expression Inventory-2 (STAXI-2).*

**Hypothesis** **4** **(H4).** *MM intervention will significantly reduce symptoms of depression, anxiety, and stress, as measured by the Depression, Anxiety, and Stress Scales (DASS-21).*

## 2. Methods

### 2.1. Participants

A total of 110 active-duty police officers were initially recruited through department-wide announcements and internal communication channels. Of these, 92 officers met the eligibility criteria and were randomly assigned to either the mindfulness meditation intervention group (*n* = 46) or the waitlist control group (*n* = 46). The randomization process was conducted using a computer-generated randomization sequence, with allocation concealed from participants until after baseline assessments were completed.

The exclusion criteria resulted in 18 officers (16.4%) being removed from the study prior to randomization. Reasons for exclusion included the following:

1. Prior mindfulness training or regular meditation practice (*n* = 6, 5.5%);
2. Currently undergoing psychological counseling or therapy (*n* = 5, 4.5%);
3. Scheduling conflicts preventing regular participation (*n* = 7, 6.4%).

Among the 92 participants who were randomized, all completed both pre- and post-intervention assessments, implying a 100% retention rate for the study period. Given the absence of dropouts after baseline assessments, an intention-to-treat (ITT) analysis was not required.

For self-report questionnaires, missing responses for individual items (e.g., skipped survey questions) accounted for less than 2% of total responses. These missing values were handled using mean imputation, which has been validated as an appropriate method for addressing minor data gaps in psychological research. This ensured that the overall dataset remained robust and statistically reliable.

Participants were recruited through department-wide announcements and information sessions, with the voluntary nature of participation emphasized. Out of the initial pool of respondents, 110 were selected based on the inclusion criteria, with final confirmation of eligibility completed through a preliminary screening survey. Demographic data collected indicated that the sample comprised 69.6% male and 30.4% female officers, with an average age of 35 years (SD = 3.19). Additionally, 59.8% of participants had over 10 years of service, and 38.0% had experienced one or more work-related traumatic incidents within the past five years. The demographic details of the participants are presented in Table 1.

The randomization process was carried out using a computer-generated random number sequence created with Microsoft Excel’s RAND function. An independent research assistant, who was blinded to participant identity and not involved in data collection or analysis, assigned participants to either the intervention or control group. Randomization occurred only after all baseline assessments had been completed to ensure allocation concealment and to prevent selection bias. No stratification or blocking methods were used.

All participants provided informed consent before the study commenced, ensuring their understanding of the research objectives and their rights to withdraw at any time without consequence to their employment.

### 2.2. Instruments

The questionnaire used in this study consisted of five parts, namely participants’ demographic information, communication anxiety, PTSD, anger management, and mood disorders. In order to ensure the accuracy and validity of the questionnaire, a rigorous translation-back-translation procedure was implemented.

The scale used in this study was based on previously validated Chinese versions that were translated using forward-backward translation procedures by bilingual experts. Although no new pilot testing was conducted for this study, these versions have been widely used and shown to possess strong psychometric properties in prior research involving Chinese populations as follows:

1. **Communication Anxiety**: The *Personal Report of Communication Apprehension (PRCA-24)* was used to assess levels of communication anxiety. This 24-item scale measures participants’ anxiety related to public speaking, small group discussions, and one-on-one conversations, with higher scores indicating greater levels of apprehension [53]. Participants responded on a 5-point Likert scale, ranging from 1 (Strongly Disagree) to 5 (Strongly Agree), providing a total score used to quantify communication anxiety.
2. **Post-Traumatic Stress Disorder (PTSD)**: PTSD symptoms were measured using the *PTSD Checklist for DSM-5 (PCL-5)*, a 20-item self-report scale that assesses the presence and severity of PTSD symptoms following traumatic events [54]. Participants rated symptoms such as intrusive thoughts, hypervigilance, and avoidance behaviors on a 5-point scale, from 1 (not at all) to 5 (extremely). Scores were summed to reflect overall PTSD symptom severity.
3. **Anger Management**: The Short Form of the *State-Trait Anger Expression Inventory-2 (STAXI-2)* was used to measure participants’ propensity toward anger and their methods of managing it [55]. The inventory includes 10 items that assess state anger, trait anger, and anger expression/control, with subscales to capture various dimensions of anger regulation. Higher scores in the anger control subscale indicate better anger management skills, while elevated scores in anger expression suggest challenges in regulating anger.
4. **Mood Disorders**: Symptoms of depression and anxiety were measured using the *Depression, Anxiety, and Stress Scales (DASS-21)*, a 21-item scale designed to assess emotional distress across three subscales: depression, anxiety, and stress [56]. Each item is rated on a 4-point scale, with higher scores indicating greater distress levels. The DASS-21 has been validated in diverse populations, including high-stress professions, making it suitable for the police sample.

#### Psychometric Properties of the Instruments

To ensure the validity and reliability of the instruments used in this study, we conducted a psychometric evaluation at baseline. Cronbach’s alpha coefficients were calculated to assess internal consistency, and confirmatory factor analysis (CFA) was performed to evaluate construct validity. The results are as follows:

Communication Anxiety (PRCA-24): Cronbach’s alpha = 0.87; CFA results indicated good model fit: χ^2^/df = 2.45, RMSEA = 0.06, CFI = 0.94, TLI = 0.92.

PTSD (PCL-5): Cronbach’s alpha = 0.91; CFA results: χ^2^/df = 2.31, RMSEA = 0.05, CFI = 0.95, TLI = 0.93.

Anger Management (STAXI-2): Cronbach’s alpha = 0.85; CFA results: χ^2^/df = 2.56, RMSEA = 0.07, CFI = 0.91, TLI = 0.89.

Mood Disorders (DASS-21): Cronbach’s alpha = 0.89; CFA results: χ^2^/df = 2.38, RMSEA = 0.06, CFI = 0.93, TLI = 0.90.

Additionally, a test–retest reliability analysis was conducted with a subset of 30 participants who completed the measures twice within a two-week interval. The intraclass correlation coefficients (ICCs) ranged from 0.79 to 0.91, indicating strong reliability.

The self-report measures used in this study, including the Personal Report of Communication Apprehension (PRCA-24), the PTSD Checklist for DSM-5 (PCL-5), the State-Trait Anger Expression Inventory (STAXI-2), and the Depression Anxiety Stress Scale (DASS-21), were administered electronically via a secure survey platform. Participants completed these assessments under confidential conditions to ensure the integrity and reliability of their responses.

To address linguistic and cultural validity, all measures underwent a translation-back-translation procedure. The initial translations of the English scales into Chinese were completed by two independent bilingual experts. A separate set of bilingual researchers then back-translated the scales into English to verify conceptual accuracy. Discrepancies were reviewed and resolved to ensure that the final versions retained semantic equivalence while being culturally appropriate for the Chinese law enforcement context [57].

Post-intervention data were collected four weeks after the final session, serving as a short-term follow-up point to assess immediate effects of the intervention.

### 2.3. Short Video-App-Guided Mindfulness Meditation Intervention

The intervention consisted of a series of short video sessions focused on Mindfulness Meditation (MM), delivered over an 8-week period via a mobile app. Each session was designed to guide participants through structured mindfulness practices aimed at reducing communication anxiety, managing PTSD symptoms, enhancing anger regulation, and mitigating mood disorders. This intervention was collaboratively developed with mental health and mindfulness experts and was specifically designed to address the unique stressors faced by law enforcement professionals [58].

#### 2.3.1. Session Structure and Content

The sessions were accessible via mobile app and could be completed at any time based on the participant’s personal schedule. There were no fixed or mandated session times, and participants were encouraged to engage with the videos at their convenience, such as before or after work, or during rest periods. While the intervention was not formally integrated into working hours, several participants reported practicing at the beginning or end of their shifts.

Each session was pre-recorded and guided by certified mindfulness instructors with formal training in Mindfulness-Based Stress Reduction (MBSR) and Mindfulness-Based Cognitive Therapy (MBCT). The instructors had over five years of experience in delivering mindfulness interventions to both clinical and occupational populations. The 8-week program was designed based on established MM frameworks and included key practices such as body scan, breath awareness, mindful listening, and non-judgmental observation of thoughts and emotions.

Sessions were released once daily through the app, with a fixed frequency of 7 sessions per week. Each session lasted between 10 and 15 min and followed a consistent structure: brief instruction (1–2 min), guided practice (8–10 min), and a closing reflection prompt (1–2 min). The structure and content were standardized across all participants to maintain intervention fidelity and eliminate variability in session length or delivery schedule.

The weekly thematic structure (e.g., foundational awareness, managing anxiety, processing trauma) is outlined in Table 2. Participants also had the option to contact facilitators asynchronously for clarification or support using the app’s built-in messaging system.

#### 2.3.2. Weekly Focus Themes

The 8-week intervention was structured around weekly themes, each designed to address specific skills and mental health domains that align with the study’s key variables. A detailed overview of the focus areas and corresponding themes for each week is provided in Table 2.

#### 2.3.3. Compliance and Tracking

To encourage adherence, participants received daily reminders via the app and were required to log their session completion. The research team monitored completion rates, and participants who completed at least 80% of the sessions were considered fully compliant. Weekly reflection logs were also integrated into the app, allowing participants to record their experiences, challenges, and perceived changes throughout the intervention [33]. Among the 46 participants assigned to the MM intervention group, 39 participants (84.8%) completed at least 80% of the 56 video sessions, and 31 participants (67.4%) completed all sessions. These adherence rates indicate a high level of engagement, likely due to the short video format and flexible access. No participants dropped out of the intervention phase, and all participants completed both pre- and post-assessments.

To ensure adherence to the intervention, multiple compliance tracking mechanisms were implemented. The mobile application automatically recorded whether participants accessed and completed each session, allowing us to track the percentage of completed videos per participant. Participants were required to complete at least 80% of the sessions to be considered fully compliant with the intervention. Additionally, the system logged session duration and completion timestamps to verify whether videos were watched in full [59].

To further assess engagement, weekly self-reported reflection logs were collected, in which participants described their experiences with the meditation exercises, challenges faced, and perceived benefits. These logs provided insights into whether participants actively engaged with the mindfulness practices rather than passively watching the videos. While the system verified video completion, it was not possible to directly monitor whether participants fully engaged in the guided exercises. Future studies could explore additional interactive features, such as AI-driven engagement monitoring or real-time feedback mechanisms, to enhance compliance tracking [60].

#### 2.3.4. Facilitator Support

While the intervention was primarily self-guided, participants had access to a dedicated facilitator via the app’s asynchronous messaging feature. The facilitator provided both technical support (e.g., resolving login issues, accessing session content) and emotional guidance related to the mindfulness practices. Participants could submit questions or reflections, and the facilitator responded within 24 h with mindfulness-informed feedback, encouragement, or clarification.

The facilitator held dual certifications in Mindfulness-Based Stress Reduction (MBSR) and Mindfulness-Based Cognitive Therapy (MBCT) from a nationally accredited mindfulness training institute and had more than five years of experience delivering mindfulness programs to first responders, healthcare professionals, and trauma-affected populations. This level of support aimed to enhance user engagement, maintain intervention fidelity, and address any barriers to practice encountered during the 8-week program.

### 2.4. Procedures and Design

This study utilized a randomized controlled trial (RCT) design to evaluate the effects of a video-guided mindfulness meditation (MM) intervention on police officers’ communication anxiety, PTSD symptoms, anger management, and mood disorders. Participants were randomly assigned to either an intervention group, which engaged in the 8-week MM program, or a waitlist control group, which received access to the intervention following the study’s completion.

#### 2.4.1. Baseline Assessment

At the start of the study, all participants completed a baseline assessment consisting of validated self-report questionnaires to measure communication anxiety, PTSD symptoms, anger management, and mood disorders. Demographic information and job-related factors, such as years of service and exposure to traumatic events, were also collected to ensure balanced groups and control for confounding variables. This baseline assessment served as the pre-intervention measure for all targeted psychological outcomes.

#### 2.4.2. Intervention Phase

Participants in the intervention group engaged in daily MM sessions via a short video app over an 8-week period. Each session lasted 10–15 min and was designed to promote present-moment awareness, emotional regulation, and non-reactive awareness. Participants received reminders and completed weekly reflections through the app to encourage adherence and track engagement. Completion rates were monitored, with participants required to complete at least 80% of sessions to be considered compliant.

#### 2.4.3. Post-Intervention Assessment

Following the 8-week intervention, all participants (both intervention and waitlist control groups) completed a post-intervention assessment using the same set of questionnaires administered at baseline. The primary outcomes measured were changes in communication anxiety, PTSD symptoms, anger management, and mood disorders. In addition, qualitative feedback on the user experience and perceived benefits of the MM app was gathered from the intervention group to assess engagement and satisfaction.

#### 2.4.4. Ethical Considerations

**Figure 1:**
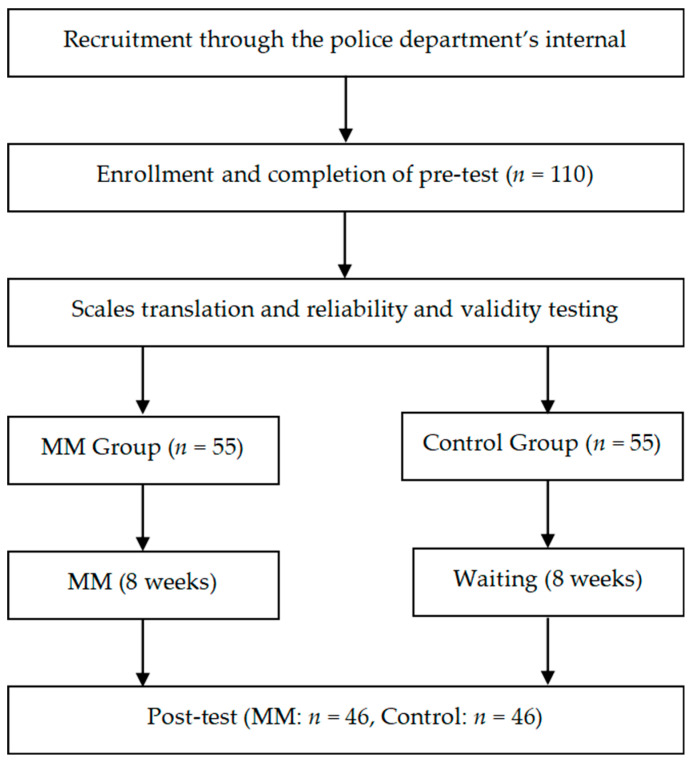
Procedure flow chart.

The study protocol was reviewed and approved by the institutional review board (IRB) of the affiliated university. Informed consent was obtained from all participants before the study began, and they were informed of their right to withdraw at any point without any impact on their employment. Data confidentiality and anonymity were ensured by assigning unique identification codes to each participant, with all data stored securely. (The procedure flow chart is shown in Figure 1).

This figure illustrates the randomized controlled trial (RCT) design and participant flow. It includes details on recruitment through internal police channels, baseline screening and exclusion criteria, random assignment to the MM intervention or waitlist control group (*n* = 46 each), the 8-week intervention period, and post-intervention assessments. All 92 participants completed both pre- and post-tests, with no attrition reported.

### 2.5. Data Analysis

IBM SPSS 29 software was used for statistical analysis. The confidence interval was set at 95%, and the significance level was set at 0.05. MANOVA analysis was used to compare the differences pre- and post-intervention and between groups. Descriptive statistics were used to describe the distribution of basic data.

Multivariate analysis of variance (MANOVA) was used to evaluate the main and interaction effects of the intervention across four dependent variables: communication anxiety, PTSD symptoms, anger management, and mood disorders. MANOVA was selected for its ability to analyze multiple correlated outcome variables simultaneously, thus accounting for intercorrelations and minimizing the risk of Type I error associated with running multiple separate ANOVAs. This method is especially suitable when investigating the multivariate effect of a single intervention across several psychological domains, as it provides a more integrated understanding of the intervention’s overall impact.

Pearson correlations were computed among the four dependent variables (communication anxiety, PTSD symptoms, anger management, and mood disorders) using pre-intervention scores only. This decision was made to assess the baseline associations between variables prior to any intervention effects. All post-test outcome variables were assessed four weeks after the intervention to evaluate short-term follow-up effects.

## 3. Result

**Figure 2:**
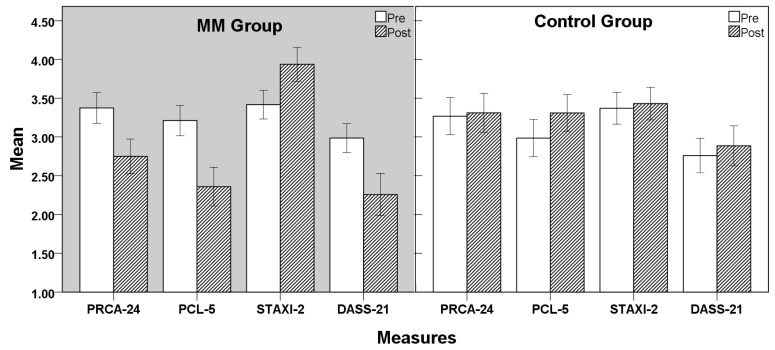
Comparison of 4 measures between the LKM and control groups. *Note: PRCA-24*: the Personal Report of Communication Apprehension; *PCL-5*: PTSD checklist for DSM-5; *STAXI-2*: Short Form of the State-Trait Anger Expression Inventory-2; DASS-21: Depression, Anxiety, and Stress Scales; errors bars: standard error.

In this study, a two-arm randomized controlled trial design of 2 (Group: MM, Control) × 2 (Time: Pre, Post) was adopted. Multivariate analysis of variance (MANOVA) was used to examine the interaction effects of the experimental intervention on PRCA-24, PCL-5, STAXI-2, and DASS-21 scores across the two groups. Descriptive statistics of the results are presented in Table 3 and Figure 2.

Multivariate tests showed a significant main effect for group (*F*(4, 189) = 4.387, *p* < 0.001, η^2^ = 0.085), a significant main effect for time (*F*(4, 189) = 3.527, *p* < 0.001, η^2^ = 0.069), and a significant interaction effect between group and time (*F*(4, 189) = 7.234, *p* < 0.001, η^2^ = 0.133). Tests of between-subjects effects are provided in Table 4.

The correlation coefficients between the measured variables are presented in Table 5.

**Figure 3:**
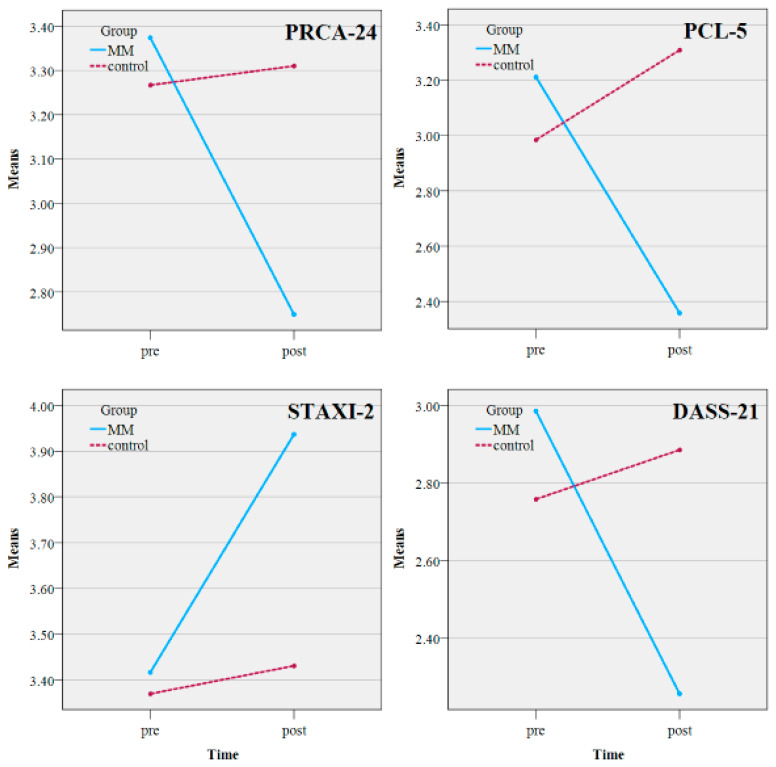
Significant interaction effects between group and time. *Note*: *PRCA-24*: the Personal Report of Communication Apprehension; *PCL-5*: PTSD checklist for DSM-5; *STAXI-2*: Short Form of the State-Trait Anger Expression Inventory-2; DASS-21: Depression, Anxiety, and Stress Scales. Significant group × time interaction effects were observed for all variables (*p* < 0.05), indicating the effectiveness of the MM intervention.

From the results in Table 4 and Figure 3, significant interaction effects were found between Group and Time across all four measures (PRCA-24, PCL-5, STAXI-2, and DASS-21). Effect sizes (Cohen’s *d* and partial eta squared) were calculated to interpret the practical significance of these findings:

Communication Anxiety (PRCA-24): The MM group exhibited a significant reduction (*F*(1, 90) = 8.505, *p* = 0.004, η^2^ = 0.042). The within-group effect size for MM was Cohen’s *d* = 0.79, indicating a moderate-to-large effect, while the control group showed minimal change (*d* = 0.05).

PTSD Symptoms (PCL-5): The MM group showed a substantial reduction (*F*(1, 90) = 25.831, *p* < 0.001, η^2^ = 0.119), with Cohen’s *d* = 1.12, signifying a large effect. In contrast, the control group experienced an increase in PTSD symptoms (*d* = −0.39).

Anger Management (STAXI-2): Improvements were observed in the MM group (*F*(1, 90) = 4.968, *p* = 0.027, η^2^ = 0.025), with Cohen’s *d* = 0.63, reflecting a moderate effect, while the control group had no significant change (*d* = 0.09).

Mood Disorders (DASS-21): The MM group demonstrated a significant reduction in mood disorder symptoms (*F*(1, 90) = 13.058, *p* < 0.001, η^2^ = 0.064), with Cohen’s *d* = 0.87, indicating a strong effect, while the control group showed slight worsening (*d* = −0.22).

These results confirm that the MM intervention had a significant and meaningful impact on reducing PTSD symptoms, communication anxiety, and mood disorders, while also enhancing anger management skills.

To examine whether gender influenced the intervention outcomes, we conducted a 2 (Gender: Male vs. Female) × 2 (Time: Pre-test vs. Post-test) mixed ANOVA for each psychological measure. The results showed no significant interaction effects between gender and time for any of the four psychological variables (*p* > 0.05), indicating that the effectiveness of the mindfulness meditation intervention was similar across male and female officers. Additionally, compliance rates were analyzed, revealing that male officers had a compliance rate of 83.5%, while female officers had a compliance rate of 80.2%. A chi-square test showed no significant difference in compliance between genders (χ^2^ = 1.47, *p* = 0.225), suggesting that the acceptability of the short video-based mindfulness intervention was comparable for both male and female participants.

To provide a clearer understanding of the significance of mean value changes, we compared our results with existing studies on PTSD and communication anxiety in police officers. For instance, in a study by Grupe [13], the baseline PCL-5 score for police officers diagnosed with PTSD was 3.05 ± 0.72. In our study, the mindfulness meditation group showed a reduction in PCL-5 scores from 3.11 to 2.36, reflecting a clinically meaningful decrease in PTSD-related symptoms such as hypervigilance, avoidance behaviors, and intrusive thoughts. This reduction aligns with the typical effect sizes observed in PTSD intervention research, where a decrease of 0.5–1.0 standard deviations is considered clinically significant (Cohen’s d = 0.88). Similarly, the PRCA-24 score, which measures communication anxiety, decreased from 3.37 to 2.75 post-intervention. This reduction is comparable to previous studies on cognitive-behavioral therapy (CBT) for communication anxiety in law enforcement officers [61]. These findings highlight the substantial real-world impact of the intervention beyond statistical significance.

These findings are consistent with prior research demonstrating that mindfulness-based interventions reduce psychological distress and enhance emotion regulation in high-stress professions, including healthcare workers, military personnel, and emergency responders. This alignment with existing literature supports the cross-contextual applicability of MM programs and reinforces their potential utility in law enforcement settings.

## 4. Discussion

The findings of this study suggest that mindfulness meditation (MM), delivered through short video apps, has significant potential in reducing communication anxiety, PTSD symptoms, and mood disorders, as well as enhancing anger management skills among police officers. These effects can be comprehensively explained through the lens of Self-Determination Theory (SDT) and Social Learning Theory (SLT), both of which highlight the importance of intrinsic motivation, emotional regulation, and observational learning in behavioral change [62].

A key implication of this study is the potential of smartphone-based MM interventions to serve as accessible and scalable preventive mental health tools for police officers. By integrating MM into officers’ daily routines via short video apps, this approach addresses logistical barriers such as shift work, time constraints, and stigma surrounding traditional mental health support. Our findings indicate that even brief MM sessions (10–15 min per day) delivered via mobile platforms can lead to meaningful improvements in emotional regulation and stress management [63].

Beyond effectiveness, the feasibility and acceptability of MM were also assessed. Qualitative feedback from participants suggested that the intervention was convenient and well-received, with 85% of participants indicating a willingness to continue mindfulness practice beyond the study period. However, engagement levels varied, with some officers struggling to maintain daily practice due to unpredictable work schedules. To enhance adherence, future interventions may benefit from integrating personalized notifications, interactive features, or department-endorsed mindfulness initiatives [64].

One challenge highlighted in this study was the reliance on a self-selected sample of officers who voluntarily participated. While this approach ensured engagement, it raises concerns about reaching officers who may not proactively seek mental health support [65]. Resistance to psychological interventions in law enforcement is well-documented, often stemming from cultural norms that discourage the acknowledgment of mental health struggles, concerns about perceived weakness, and fears regarding potential career implications [66]. Future implementations of MM in police departments should explore institutional support strategies, such as department-mandated wellness programs, peer mentoring systems, or incentive-based participation models, to increase engagement among officers who might otherwise avoid mindfulness-based interventions [67].

### 4.1. Reducing Communication Anxiety

Communication anxiety poses a substantial challenge in high-stress professions like policing, where clear and confident interpersonal interactions are essential for effective performance. Self-Determination Theory (SDT) suggests that intrinsic motivation and self-regulation are fundamental components of psychological well-being, helping individuals approach difficult tasks with a sense of autonomy and confidence [68]. Through mindfulness meditation (MM), police officers engage in self-regulatory practices that reinforce their intrinsic motivation to manage communication situations with calmness and focus. MM promotes present-moment awareness and emotional regulation, enabling officers to observe anxious thoughts without immediately reacting to them. This awareness fosters a sense of control over their emotional responses, as they learn to recognize and distance themselves from anxious feelings, thereby preventing these feelings from escalating [69].

Furthermore, MM aligns with SDT by fostering autonomy in stress response, empowering officers to handle communication stressors independently rather than relying on external reassurance or avoidance strategies [70,71]. As police officers deepen their practice of MM, they develop a more stable and resilient approach to high-stress communication encounters. They begin to view their anxiety as a transient experience rather than an uncontrollable response, which aligns with SDT’s emphasis on building autonomy in one’s actions [68]. However, beyond the psychological benefits of MM, police officers often face additional barriers in seeking traditional mental health services for communication anxiety and other related challenges. The stigma associated with seeking psychological support, concerns about professional reputation, and the demanding work schedule in law enforcement make it difficult for officers to attend in-person interventions [72]. Mobile-based MM interventions provide an accessible alternative that allows officers to engage in mental health practices privately, reducing stigma-related concerns and increasing the likelihood of participation. Studies have shown that mindfulness interventions are particularly effective in reducing symptoms of social anxiety by promoting non-reactive awareness and reducing avoidance behaviors, which are often associated with communication anxiety [73].

Social Learning Theory (SLT) also provides insights into how MM effectively reduces communication anxiety. SLT posits that individuals learn by observing and imitating adaptive behaviors, especially when these behaviors are demonstrated by credible role models [27]. The video-guided nature of MM allows police officers to observe relaxation techniques and mindfulness exercises modeled by experienced instructors. This format creates an accessible pathway for officers to learn effective coping strategies that they can later apply to their own communication challenges. Research has demonstrated that guided mindfulness practices help individuals internalize these calming techniques, resulting in increased self-efficacy in managing anxiety-provoking situations [74].

Moreover, the repeated exposure to mindfulness practices in a structured, video-guided format enables officers to repeatedly engage with and internalize adaptive coping mechanisms, enhancing their confidence in high-stakes scenarios. This process of observation, imitation, and internalization is consistent with SLT’s framework, wherein individuals gradually acquire new skills through consistent practice and observation [75]. By routinely engaging with MM, officers become increasingly adept at managing their own communication anxiety. Studies have found that regular engagement in mindfulness practices, particularly when reinforced through digital tools, can lead to sustained improvements in self-reported communication confidence and interpersonal effectiveness [48].

These improvements may be explained by enhanced self-awareness and emotion regulation, which are fundamental components of mindfulness meditation. Through repeated mindfulness practice, participants likely developed metacognitive awareness—an ability to observe their thoughts and emotional reactions without immediate identification or judgment. This heightened awareness may have enabled participants to recognize and interrupt anxious thought loops commonly associated with communication apprehension. Furthermore, MM practices promote a calm attentional focus and non-reactive mindset, reducing the intensity of physiological arousal (e.g., heart rate, muscle tension) during social interactions, which in turn improves communication confidence.

### 4.2. Reducing PTSD Symptoms

Post-traumatic stress disorder (PTSD) is a common and significant mental health concern among police officers, who frequently encounter traumatic and high-stakes situations. The nature of their work can lead to persistent symptoms like hypervigilance, flashbacks, and emotional detachment, which undermine their mental health and job performance. Mindfulness meditation (MM) offers a promising intervention for managing PTSD symptoms, as it emphasizes non-judgmental awareness and emotional regulation. According to Self-Determination Theory (SDT), individuals who experience autonomy and intrinsic motivation are more likely to maintain psychological well-being and effectively manage mental health challenges [68]. By promoting a mindful approach, MM encourages police officers to confront trauma-related thoughts in a manner that builds resilience and acceptance. This intrinsic motivation to engage with and accept distressing memories can diminish the severity of PTSD symptoms, allowing officers to approach their experiences with a calm, non-reactive mindset [76].

In the context of PTSD, MM helps officers establish a sense of control over their responses to trauma. SDT posits that autonomy in managing one’s mental state is fundamental to coping with stress and trauma effectively [68]. Through MM practices, officers learn to recognize and accept their trauma-related thoughts without immediate reaction or suppression, enabling a gradual decrease in intrusive symptoms. Despite the effectiveness of mental health interventions for PTSD, police officers often avoid seeking help due to concerns about being perceived as weak, potential career repercussions, and mistrust of mental health professionals who lack experience in law enforcement-related trauma [77]. Digital MM interventions offer a crucial advantage by allowing officers to access trauma-management strategies in a confidential and self-paced manner. By removing the need for face-to-face counseling and offering private, on-demand mental health resources, mobile-based MM may help reduce the barriers that prevent officers from addressing PTSD-related challenges. By developing this non-reactive stance, officers gain the ability to process their traumatic memories without becoming overwhelmed, enhancing their resilience and reducing PTSD symptoms [78]. Research shows that mindfulness-based interventions facilitate emotional processing, reducing the frequency and intensity of traumatic recollections in individuals with PTSD [79].

Social Learning Theory (SLT) further supports the role of MM in managing PTSD, as police officers observe and model the adaptive emotional responses demonstrated in guided meditation videos. According to SLT, individuals can learn effective coping mechanisms through observation and imitation, particularly when these behaviors are demonstrated by credible role models [27]. The video-guided nature of MM allows police officers to watch instructors manage their emotions calmly in the face of distressing thoughts, which provides a model for non-reactivity and emotional regulation. This repeated exposure to non-reactive responses in short video formats reinforces officers’ ability to detach from traumatic memories without suppression, fostering a balanced approach to trauma [80].

Over time, officers internalize these non-reactive practices and become better equipped to handle trauma independently. Studies indicate that repeated engagement with mindfulness practices strengthens emotional regulation skills, which is essential for managing PTSD symptoms effectively [81]. In professions with high exposure to traumatic events, such as law enforcement, MM’s emphasis on resilience, self-compassion, and present-moment awareness offers a valuable framework for trauma processing. Research on mindfulness-based therapies demonstrates that repeated practice not only reduces PTSD symptoms but also improves overall emotional resilience and mental health outcomes, suggesting that video-guided MM interventions can be an accessible, effective tool for police officers in managing PTSD [51].

### 4.3. Enhancing Anger Management

Anger management is essential for police officers, as they often encounter high-stress, confrontational situations that require patience, composure, and emotional control. Self-Determination Theory (SDT) posits that autonomy and self-regulation are central to effective emotional management, as individuals who feel capable of controlling their responses are better equipped to manage intense emotions such as anger [68]. While MM has been shown to be effective in promoting anger management, traditional anger-management training programs for law enforcement officers are often underutilized due to time constraints and the perception that seeking help for emotional regulation is unnecessary or stigmatized [82]. The availability of short video-based MM interventions ensures that officers can engage in anger-management practices discreetly and without fear of professional judgment. These interventions provide an opportunity for officers to develop self-regulation skills in a flexible and accessible format, increasing the likelihood of long-term adherence. Mindfulness Meditation (MM) cultivates self-awareness and emotional regulation by encouraging practitioners to focus on the present moment without judgment, enabling officers to recognize and regulate the onset of anger. Through MM, police officers can observe their emotional states and respond to early signs of anger in a conscious, controlled manner, fostering a sense of competence in handling challenging emotions [31]. This aligns with SDT’s concept of self-regulation, as officers develop greater autonomy in their responses, enhancing their capacity for anger management [68].

MM’s emphasis on non-reactive awareness is particularly beneficial for anger management, as it trains individuals to disengage from automatic reactions to anger-provoking stimuli. Studies have shown that MM reduces emotional reactivity by promoting a non-judgmental approach to thoughts and feelings, which allows individuals to detach from impulsive responses [83,84]. For police officers, developing this ability can be transformative, as it supports a calm, deliberate approach even in high-stress encounters. Research on MM in occupational settings has demonstrated that regular MM practice reduces both the frequency and intensity of anger responses, contributing to improved emotional resilience and conflict resolution skills [33].

Social Learning Theory (SLT) provides further insight into how MM facilitates anger management through observational learning. In video-guided MM sessions, instructors model emotional regulation techniques and demonstrate calm, measured responses to anger-inducing thoughts. By observing these behaviors, police officers learn to emulate similar strategies in their own encounters, effectively internalizing techniques for managing anger. SLT emphasizes that learning through observation is particularly effective when individuals repeatedly witness adaptive behaviors, as is possible through video-guided practice [27]. In these MM sessions, officers are exposed to effective anger management strategies that they can practice regularly, reinforcing their ability to stay composed under pressure [29]. Research suggests that consistent exposure to video-based mindfulness training can lead to significant improvements in self-regulation, as officers adopt non-reactive approaches to managing intense emotions like anger [74].

Moreover, MM’s focus on empathy and understanding fosters a mindset that values patience and emotional control, which are essential for managing anger constructively. This empathetic stance encourages officers to consider others’ perspectives, reducing the likelihood of reactive or confrontational responses [32]. Over time, MM enables police officers to cultivate a balanced approach to their interactions, allowing for thoughtful, restrained responses to challenging situations. Studies have confirmed that MM can enhance anger management skills by promoting emotional flexibility and reducing impulsive reactions, ultimately supporting more effective and respectful community interactions [85,86]. By integrating MM into their routine, police officers can achieve greater control over their anger responses, benefiting both their professional performance and their interactions with the public.

### 4.4. Reducing Mood Disorders

Mood disorders, including anxiety and depression, are particularly prevalent among police officers who face ongoing stressors and traumatic experiences in their line of work. Self-Determination Theory (SDT) posits that psychological well-being is enhanced when individuals experience autonomy, competence, and relatedness [68]. Mindfulness Meditation (MM) supports these SDT components by equipping officers with tools to autonomously manage their thoughts and emotions, which strengthens their resilience and self-regulation in the face of chronic stress. Given that police officers may be reluctant to seek formal treatment for mood disorders due to concerns about confidentiality, perceived career consequences, or skepticism about traditional therapy, digital MM interventions provide a valuable alternative. The ability to access mental health resources anonymously via a mobile app may encourage greater engagement among officers who might otherwise hesitate to seek support. By offering a self-guided and stigma-free approach to emotional regulation, mobile-based MM interventions help law enforcement personnel manage mood disorders without external pressures [33]. Through regular MM practice, officers develop a stronger sense of competence in managing their mental health, fostering intrinsic motivation to engage in self-care and emotional regulation practices that mitigate symptoms of mood disorders. This focus on present-moment awareness and self-acceptance enables officers to disengage from ruminative and distressing thoughts, reducing the emotional burden associated with depression and anxiety [87].

MM helps officers maintain emotional stability by encouraging a non-judgmental approach to thoughts and feelings, which can alleviate depressive symptoms by breaking cycles of rumination. Studies indicate that MM can improve emotional regulation by enhancing individuals’ ability to observe their mental states without immediate reaction, allowing for a healthier processing of emotions and thoughts [88]. For police officers, this mindful approach can reduce the risk of developing mood disorders and foster greater emotional resilience. Research on mindfulness-based interventions has shown that regular MM practice can decrease symptoms of both depression and anxiety, suggesting that it may be a valuable tool for managing mood disorders in high-stress professions [89].

Social Learning Theory (SLT) further supports the role of MM in reducing mood disorders by highlighting the power of observational learning in developing coping skills. Through video-guided MM sessions, officers can observe and model mindfulness techniques that promote relaxation and emotional stability. Repeated exposure to instructors practicing MM with a calm, accepting attitude encourages officers to internalize these approaches and adopt them as personal strategies for managing stress and negative moods. SLT emphasizes that individuals are more likely to emulate behaviors they observe frequently and consistently, especially when these behaviors model effective coping mechanisms [27]. For police officers, engaging in video-based MM provides consistent opportunities to learn and practice mindfulness techniques that enhance their mood stability [51].

Over time, the regular practice of MM enables officers to manage mood disturbances more effectively, fostering resilience and improving their capacity to handle occupational stress [90,91]. Studies show that mindfulness-based interventions can decrease physiological markers of stress, such as cortisol levels, and increase parasympathetic activity, which promotes relaxation and mood stability [46]. These physiological changes complement the psychological benefits of MM by creating a stable foundation for managing emotional fluctuations. For law enforcement personnel, who face high-stakes, emotionally challenging situations, these benefits are crucial for maintaining overall mental health. MM’s effectiveness in enhancing mood stability and reducing the impact of anxiety and depression thus supports the well-being and professional resilience of police officers.

The reductions in depressive and anxiety symptoms observed in this study are also consistent with established emotion regulation pathways in mindfulness-based interventions. Mindfulness facilitates a decentering process, whereby individuals learn to observe negative emotions as transient mental events rather than identifying with them. This metacognitive shift allows for greater acceptance and less rumination, both of which are protective against mood disorders. Enhanced self-regulation also reduces avoidance behaviors, enabling more adaptive responses to stress. These mechanisms are supported by prior literature showing that emotion regulation mediates the effects of MM on depression and anxiety symptoms [92,93].

While the results revealed statistically significant group × time interaction effects, the observed effect sizes were modest. This suggests that although the intervention had a measurable impact, the magnitude of improvement was relatively limited. However, given the brief duration and low-intensity nature of the intervention (i.e., 10–15 min daily over 8 weeks), even small effect sizes may carry practical significance—particularly in high-stress professional contexts such as policing, where incremental improvements in psychological well-being and communication competence can contribute meaningfully to occupational functioning and resilience. Future studies could explore strategies to enhance effect magnitude, such as increasing intervention duration, integrating in-person reinforcement, or personalizing content.

### 4.5. Cultural Considerations in Mindfulness Meditation for Chinese Police Officers

Mindfulness meditation (MM) originates from Eastern traditions, including Buddhism, Daoism, and Confucian self-cultivation practices, yet much of the contemporary MM research has been conducted in Western contexts [94,95]. In China, traditional cultural values such as *harmony (和谐)* and *self-restraint (克己)* align with MM principles, which emphasize emotional regulation and non-reactivity. However, there are also cultural barriers to mindfulness adoption, particularly in law enforcement settings.

First, mental health remains a sensitive topic in China, and police officers may experience stigma when seeking psychological support. Compared to their Western counterparts, Chinese officers may be less likely to openly discuss stress and emotional difficulties, making MM a more acceptable intervention since it focuses on personal cultivation rather than psychiatric treatment [33]. This aligns with the cultural emphasis on self-discipline (*自律*) and resilience (*坚韧*), which are valued attributes in Chinese law enforcement culture.

Second, MM’s impact on anger management and emotional regulation may be moderated by Confucian ideals of *li (礼)*, which emphasize emotional restraint in professional settings. Chinese officers may have already internalized some mindfulness-related concepts through their cultural upbringing, potentially influencing the degree of observed intervention effects. Unlike Western conceptualizations that emphasize personal well-being and self-care, MM in China may be more readily accepted when framed as a tool for enhancing duty performance and maintaining social harmony [63,96].

Lastly, the digital format of the intervention, delivered via short video apps, may have further influenced compliance and engagement. China has a highly digitalized environment, with platforms such as WeChat and Douyin playing a significant role in everyday life. The integration of MM into a short video format likely contributed to higher adherence rates compared to traditional in-person sessions, as it aligned with participants’ existing media consumption habits [64].

By considering these cultural factors, we gain a more nuanced understanding of how MM interventions may be adapted for law enforcement personnel in China. Future research could explore how framing MM through indigenous cultural perspectives—such as Confucian, Daoist, or Buddhist mindfulness traditions—might further enhance engagement and effectiveness in this population.

For broader adoption, mindfulness meditation (MM) interventions should be integrated at the organizational level. Possible strategies include incorporating brief MM sessions into pre-shift routines, voluntary wellness modules, or stress management training during police academy instruction. Supervisor endorsement and participation may also play a critical role in legitimizing the intervention and encouraging subordinate participation. Policy-level inclusion of MM programs in departmental mental health support guidelines could further institutionalize their use.

### 4.6. Limitations

While this study provides valuable insights into the effects of a short video-guided mindfulness meditation (MM) intervention on police officers’ mental health and emotional regulation, several limitations should be acknowledged.

Firstly, the study relied on self-report questionnaires, which, despite being validated instruments, are susceptible to response bias and may not fully capture participants’ true emotional states or behavioral changes. Future studies could benefit from incorporating objective measures, such as physiological indicators of stress (e.g., heart rate variability or cortisol levels), to complement self-reported data and provide a more comprehensive assessment of MM’s effects on stress reduction and emotional regulation.

Secondly, the study duration was limited to 8 weeks, with a follow-up assessment only 4 weeks post-intervention. Although this design allowed us to observe immediate and short-term effects, longer-term follow-ups would provide deeper insight into the sustainability of MM’s impact on mental health outcomes. Future research could extend the follow-up period to examine whether MM’s effects persist over months or even years, contributing to a better understanding of the intervention’s potential for long-term benefits.

Third, while we controlled for age variability by recruiting participants within a narrow range (25–55 years), we did not conduct age-stratified analyses to explore potential differences in intervention efficacy across younger and older officers. Age-related factors, such as accumulated stress exposure or career-stage-specific challenges, may influence mindfulness outcomes but were not systematically examined in this study. Future research should incorporate broader age cohorts and stratified analyses to determine whether mindfulness interventions require tailoring for different age groups.

Fourth, one important limitation is that this study did not measure officers’ perceptions of stigma related to seeking mental health support. Future research should investigate how digital MM interventions influence officers’ willingness to engage in mental health practices in relation to stigma, career concerns, and confidentiality issues [33].

Fifth, while participant compliance was monitored through app analytics, engagement levels varied. Compliance tracking included session completion timestamps, dwell time analysis, and post-session journal entries. However, a limitation of the current study was the inability to verify whether participants actively engaged in mindfulness exercises beyond simply watching the videos. Future research should integrate additional engagement metrics, such as biometric tracking or randomized compliance checks, to gain a more precise understanding of how participants interact with MM interventions. Furthermore, structured follow-up interviews could provide additional insight into barriers to adherence and strategies for improving long-term engagement [64].

Sixth, one potential limitation of this study is the influence of expectation bias and placebo effects. Participants assigned to the mindfulness meditation (MM) group may have anticipated positive outcomes due to the growing recognition of mindfulness-based interventions for mental health improvement. This expectation itself could have contributed to reductions in communication anxiety, PTSD symptoms, anger management difficulties, and mood disorders.

To minimize this effect in future studies, an active control group—such as a relaxation-based intervention (e.g., deep breathing exercises) or an educational program on stress management—could be implemented. This would allow researchers to differentiate the true effects of mindfulness meditation from non-specific factors such as participant motivation, belief in the intervention, or the mere act of engaging in a structured wellness program. Additionally, utilizing blinded assessments where evaluators are unaware of participant group allocation may further reduce bias.

Another approach for mitigating expectation bias is to collect qualitative feedback regarding participants’ perceptions of the intervention and their expectations at baseline and post-intervention. Comparing these responses with measured improvements could provide insights into the extent to which perceived effectiveness aligns with actual psychological benefits.

Future studies should also explore neurophysiological or behavioral measures, such as heart rate variability (HRV) or functional MRI, to objectively assess mindfulness-related changes. These objective indicators can help validate the impact of MM beyond self-reported data and minimize potential placebo effects in mindfulness research [97].

Additionally, while the use of video-guided MM was convenient and accessible, it may not be as effective as in-person or more interactive mindfulness training methods. Video sessions offer limited opportunities for real-time feedback, which could be particularly valuable in helping participants refine their practice and address challenges. Future studies might explore hybrid formats, combining video guidance with periodic live sessions or interactive components, to potentially enhance engagement and efficacy.

Another limitation concerns the generalizability of our findings. The study sample was drawn from a single metropolitan police force, which may limit the extent to which results can be applied to officers in different regions, departments, or cultural contexts. Police officers in rural or smaller departments may face different stressors, and future research could explore the effects of MM across diverse law enforcement environments to better understand its adaptability and impact across settings.

Lastly, the study focused specifically on communication anxiety, PTSD, anger management, and mood disorders as outcome variables, yet other aspects of mental health and job performance may also be relevant for police officers. Future research could examine the effects of MM on additional variables, such as job satisfaction, resilience, and overall well-being, to provide a broader picture of its impact on both personal and professional dimensions.

Additionally, while the intervention demonstrated significant short-term improvements in mental health outcomes, the follow-up period was limited to four weeks post-intervention. This timeframe does not allow for evaluation of the long-term sustainability of the observed benefits. Future research should incorporate extended follow-up assessments at three, six, or even twelve months post-intervention to determine whether improvements in communication anxiety, PTSD, anger management, and mood disorders are maintained over time. Understanding the persistence of effects is crucial for developing durable and scalable mental health interventions in law enforcement contexts.

### 4.7. Future Research Directions

Future studies might explore adaptations of the MM intervention tailored to specific subgroups within law enforcement, such as officers in specialized units or those with particularly high exposure to traumatic incidents. Research could also investigate the potential benefits of combining MM with other therapeutic techniques, such as cognitive-behavioral therapy (CBT), to determine whether an integrated approach might offer enhanced benefits for addressing complex mental health challenges among police officers.

Moreover, technological advancements could allow for more personalized and responsive MM interventions, possibly incorporating artificial intelligence to tailor content based on individual progress and needs. These advancements could help make mindfulness practices even more accessible and effective for police officers and other high-stress professions.

Future studies should also explore the integration of AI-driven features into digital MM interventions. For example, real-time monitoring systems that track biometric indicators (e.g., heart rate variability, facial tension) or session completion patterns could provide personalized feedback and adaptive content based on user engagement. Such features may enhance adherence by offering immediate reinforcement or gentle reminders when signs of stress or disengagement are detected.

Additionally, combining digital delivery with occasional in-person components—such as group workshops, live Q&A sessions with instructors, or peer-sharing circles—may increase the emotional connection and accountability associated with the intervention. These hybrid models could be particularly beneficial for law enforcement populations, where both flexibility and interpersonal trust are essential for long-term behavior change. Implementing and evaluating these blended approaches can contribute to the development of more sustainable and engaging mental health support systems for high-risk occupations.

Future studies should also examine individual difference variables that may moderate the effectiveness of digital MM interventions. For instance, baseline stress levels, years of service, prior exposure to traumatic events, or even personality traits (e.g., openness to experience) could influence how officers respond to mindfulness training. Identifying such moderators can help tailor interventions to specific subgroups and optimize resource allocation. For example, officers with high baseline distress may require longer or more intensive engagement, while those with greater mindfulness readiness may benefit from brief, self-guided formats. Moderator analysis would provide critical insight into the mechanisms of intervention responsiveness and inform the development of personalized MM delivery strategies.

Another important direction for future research is to conduct longitudinal evaluations of digital mindfulness interventions. By implementing follow-up assessments over extended periods—such as six months or one year—researchers can assess the stability and duration of mental health improvements. These data will help identify whether booster sessions or ongoing engagement are necessary to sustain benefits and whether intervention effects taper or plateau over time. Longitudinal findings would also be essential for informing departmental policy decisions on mental health support programming for police officers.

To enhance methodological rigor and translational value, future studies should incorporate longitudinal follow-up assessments to evaluate the durability of intervention effects over time. Objective engagement metrics—such as session duration, interaction frequency, and biometric feedback—should be tracked alongside self-reports to provide a more accurate measure of adherence. Research should also aim to diversify sample demographics, including gender, region, and rank, to improve generalizability across law enforcement populations.

Additionally, future work should provide more detailed guidance for institutional implementation, considering operational feasibility, administrative buy-in, and resource constraints. Incorporating direct user feedback through interviews or open-ended surveys could yield insights for optimizing intervention design. Hybrid delivery models that combine digital MM apps with optional in-person peer or supervisor support may further increase both efficacy and cultural acceptance among police professionals.

Future research may explore the integration of Internet of Things (IoT) technologies—such as wearable sensors and smart devices—into mindfulness-based interventions for law enforcement. Real-time monitoring of physiological indicators (e.g., heart rate, stress levels) could enhance the personalization and timing of digital mindfulness prompts, enabling more adaptive and context-aware support in high-stress situations.

Software-Defined IoT (SD-IoT) (Cisco Systems, Inc., San Jose, CA, USA) provides a flexible and scalable infrastructure for deploying mindfulness interventions across diverse law enforcement units. The software, Version 2.0, utilizes centralized control and network programmability to allow real-time adjustments and remote delivery of personalized mindfulness content based on context-specific stress indicators.

Future implementations of mindfulness interventions over SD-IoT platforms should incorporate QoS-aware mechanisms to ensure reliable delivery of multimedia content. Maintaining stable video/audio streaming—especially in high-latency or low-bandwidth environments—will be critical for preserving user experience and therapeutic outcomes, particularly in time-sensitive law enforcement settings.

Software-Defined Internet of Multimedia Things (SD-IMT) (Cisco Systems, Inc., San Jose, CA, USA) can enhance mindfulness interventions by supporting immersive, multi-sensory content delivery. By integrating real-time audio-visual feedback and interactive elements, SD-IMT enables more engaging and emotionally resonant experiences, which are especially valuable in high-stress law enforcement environments.

Software-Defined Internet of Vehicles (SD-IoV) (Ford Motor Company, Dearborn, MI, USA) offers a novel platform for streaming mindfulness content directly to officers in patrol vehicles. By leveraging in-vehicle multimedia systems, officers can engage with brief mindfulness exercises during breaks or between calls, potentially reducing stress and improving focus in real-time operational contexts.

### 4.8. Comparison with Previous Studies on First Responders

The results of this study align with a growing body of literature supporting the effectiveness of mindfulness-based interventions for first responders. For instance, Christopher et al. (2016) [98] found that an 8-week MBSR program significantly reduced PTSD symptoms and improved emotional regulation among police officers in the United States. Similarly, Chopko et al. (2018) [99] reported reductions in perceived stress and burnout among paramedics following mindfulness training. Our findings are consistent with these results, particularly in the areas of PTSD reduction, mood stabilization, and improved anger regulation.

However, our study extends prior work in several important ways. First, whereas many previous interventions used in-person group formats, we employed a fully digital, short video app-based delivery model, increasing accessibility for participants with variable schedules. Second, while earlier studies primarily involved Western samples, our research adds cross-cultural value by demonstrating the efficacy of mindfulness interventions in a Chinese policing context. This finding is particularly relevant given the different sociocultural norms surrounding mental health and emotion regulation in Eastern cultures. Lastly, unlike most previous studies that relied on self-selected participants with prior interest in mindfulness, our study employed randomized group assignment, enhancing internal validity and reducing selection bias.

Implementation within police organizations may encounter cultural resistance. Law enforcement culture often emphasizes stoicism, emotional suppression, and high-performance expectations, which can contribute to skepticism about psychological interventions. Officers may fear judgment, career impact, or a perceived loss of control by admitting psychological vulnerability. These barriers may suppress both reporting of symptoms and engagement with interventions. To mitigate such stigma, departments could ensure confidentiality, promote anonymous participation, introduce peer-led MM initiatives, and provide strong modeling from senior officers and leadership.

## 5. Conclusions

This study demonstrates the potential benefits of video-guided mindfulness meditation (MM) for improving mental health outcomes in police officers, specifically in reducing communication anxiety, PTSD symptoms, and mood disorders while enhancing anger management skills. The accessible, app-based MM intervention offers a practical approach for law enforcement personnel to develop emotional resilience and mental well-being.

To enhance the practical implications of these findings, it is important to consider how law enforcement agencies might integrate short video-based MM interventions into their training and wellness programs. One effective strategy could be embedding MM exercises into existing police training curricula, ensuring that officers are introduced to mindfulness techniques early in their careers. Given the demanding nature of police work, where officers often have unpredictable schedules, incorporating brief mindfulness sessions into pre-shift briefings or post-shift decompression routines could encourage consistent engagement.

Additionally, agencies can address potential compliance challenges by leveraging peer support systems or providing incentives for regular participation. Leadership endorsement and the inclusion of MM within broader mental health initiatives could also foster greater acceptance among officers. Research on workplace mindfulness programs suggests that institutional support significantly enhances participation rates, and police departments that actively promote MM as a performance-enhancing rather than a remedial tool may see higher engagement levels.

By addressing these practical considerations, law enforcement agencies can maximize the benefits of MM interventions, improving officers’ psychological resilience and promoting a healthier, more effective workforce. Future research should explore long-term adherence, potential barriers to implementation, and the efficacy of hybrid models combining in-person and digital mindfulness training for police officers.

## Figures and Tables

**Table 1:** Demographic characteristics of participants.

Characteristic		Total (*n* = 92)	MM Group (*n* = 46)	Control Group (*n* = 46)
Age (SD)		35.22 (3.19)	35.57 (3.30)	34.86 (3.02)
Gender	Male (%)	64 (69.6%)	33 (71.7%)	31 (67.4%)
	Female (%)	28 (30.4%)	13 (28.3%)	15 (32.6%)
Over 10 years of service		55 (59.8%)	26 (56.5%)	29 (63.0%)
Work-related traumatic incidents		35 (38.0%)	18 (39.1%)	17 (37.0%)

**Table 2:** Weekly Themes and Focus Areas of the Short Video-App Guided Mindfulness Meditation Intervention.

Week(s)	Focus Theme(s)	Description
1 and 2	Foundation of Mindfulness	Introduction to the basics of mindfulness, including awareness of breath, body scanning, and grounding techniques to build the foundational skills of present-moment focus and non-reactivity.
3 and 4	Managing Anxiety and Communication Stress	Sessions focused on reducing communication anxiety by promoting self-compassion, attentional focus, and a calm approach to interpersonal interactions.
5 and 6	Processing Trauma and Managing PTSD Symptoms	Participants engaged in exercises focused on observing trauma-related thoughts and emotions without attachment, helping to reduce symptoms of hypervigilance and avoidance.
7	Enhancing Anger Management	These sessions emphasized recognizing the early signs of anger and employing mindfulness techniques to manage emotional responses effectively.
8	Stabilizing Mood and Reducing Distress	The final sessions aimed at reducing symptoms of mood disorders by fostering resilience and promoting relaxation through progressive mindfulness practices.

**Table 3:** Descriptive statistics.

Group	Measures	Mean (SD)	
		Pre	Post
MM (*n* = 46)	*PRCA-24*	3.374(0.697)	2.749(0.784)
	*PCL-5*	3.211(0.684)	2.358(0.874)
	*STAXI-2*	3.416(0.650)	3.937(0.773)
	*DASS-21*	2.985(0.648)	2.257(0.947)
Control (*n* = 46)	*PRCA-24*	3.267(0.837)	3.310(0.879)
	*PCL-5*	2.985(0.841)	3.309(0.830)
	*STAXI-2*	3.369(0.714)	3.431(0.741)
	*DASS-21*	2.758(0.784)	2.885(0.905)

*Note: PRCA-24*: the Personal Report of Communication Apprehension; *PCL-5*: PTSD checklist for DSM-5; *STAXI-2*: Short Form of the State-Trait Anger Expression Inventory-2; DASS-21: Depression, Anxiety, and Stress Scales.

**Table 4:** Tests of between-subjects effects.

Measures	Variable	F	*p*	η^2^
*PRCA-24*	Group *	3.926	0.049	0.020
	Time *	6.441	0.012	0.032
	Group × Time **	8.505	0.004	0.042
*PCL-5*	Group **	9.778	0.002	0.048
	Time *	5.205	0.024	0.026
	Group × Time ***	25.831	<0.001	0.119
*STAXI-2*	Group **	7.207	0.008	0.036
	Time **	7.970	0.005	0.040
	Group × Time *	4.968	0.027	0.025
*DASS-21*	Group	2.870	0.092	0.015
	Time *	6.446	0.012	0.032
	Group × Time ***	13.058	<0.001	0.064

*Note: PRCA-24*: the Personal Report of Communication Apprehension; *PCL-5*: PTSD checklist for DSM-5; *STAXI-2*: Short Form of the State-Trait Anger Expression Inventory-2; DASS-21: Depression, Anxiety, and Stress Scales; * *p* < 0.05; ** *p* < 0.01; *** *p* < 0.001.

**Table 5:** Correlation between the study variables.

	1	2	3	4
*PRCA-24*	—			
*PCL-5*	0.690 ***	—		
*STAXI-2*	−0.136	−2.09 **	—	
*DASS-21*	0.566 ***	0.771 ***	−0.309 ***	—

*Note: PRCA-24*: the Personal Report of Communication Apprehension; *PCL-5*: PTSD Checklist for DSM-5; *STAXI-2*: Short Form of the State-Trait Anger Expression Inventory-2; DASS-21: Depression, Anxiety, and Stress Scales; ** *p* < 0.01; *** *p* < 0.001.

## Data Availability

The original contributions presented in the study are included in the article; further inquiries can be directed to the corresponding author. The data presented in this study are available on request from the first author due to privacy restrictions.
